# A Switched-Line True Time Delay Unit for Wideband Phased Arrays Using Packaged RF MEMS Switches

**DOI:** 10.3390/s25216806

**Published:** 2025-11-06

**Authors:** David W. K. Thomas, Kai Wu, Y. Jay Guo

**Affiliations:** Global Big Data Technologies Centre, University of Technology Sydney, Sydney, NSW 2007, Australia; david.w.thomas-1@student.uts.edu.au (D.W.K.T.); jay.guo@uts.edu.au (Y.J.G.)

**Keywords:** broadband beamforming, coplanar waveguide (CPW), group delay ripple, phased arrays, RF MEMS switches, switched-line architecture, true time delay unit (TDU)

## Abstract

The growing demand for wideband electronically scanned arrays (ESAs) in next-generation radar, satellite, and 5G/6G systems has renewed interest in true time delay units (TDUs) to overcome the limitations of phase-based beamforming. In parallel, recent advances in the commercial availability and reliability of packaged RF MEMS switches have enabled practical hardware implementations once considered infeasible. This paper presents the design, fabrication, and experimental validation of a broadband, 4-bit switched-line TDU using only off-the-shelf components and standard PCB processes. The unit operates from 0.4 to 6 GHz, with a total delay range of 0–413 ps, achieving an average insertion loss of 1.5 dB and delay error below 18.4 ps, resulting in a figure of merit (FOM) of 152.8 ps/dB. Measured results are reported alongside a refined switch/termination model that aligns simulations with measurements. This is among the first reported demonstrations of a complete RF MEMS-based TDU implemented entirely with commercially available components in a standard PCB-integrated implementation. These results demonstrate a practical pathway toward scalable MEMS-based TDUs for deployment in advanced beamforming systems.

## 1. Introduction

Next-generation satellite communications and broadband wireless systems increasingly require agile, wideband beamforming solutions to meet the growing demand to connect more devices wirelessly, improve coverage, and increase data rates. Electronically scanned arrays (ESAs) are a necessary enabling technology for these systems, enabling rapid, programmable beamforming with directional control and increasing data rates with spatial multiplexing. Traditional beamformers rely on phase shifters for beam steering, but this approach introduces frequency-dependent distortion: beam squint and signal dispersion degrade spatial accuracy and time-domain fidelity. These limitations are particularly pronounced in wideband systems, such as emerging 5G/6G, low Earth orbit (LEO) satellite constellations, and joint radar–communications platforms.

Replacing the phase shifters of a phased array with true time delay units (TDUs), forming a timed array, overcomes these issues by introducing frequency-independent delays, enabling precise broadband beamforming without squint or distortion [1,2]. Despite these advantages, TDUs remain underutilized in practice due to challenges in size, cost, reliability, and manufacturability. Photonic, MMIC (monolithic microwave integrated circuit), and distributed MEMS transmission line (DMTL) TDUs have each demonstrated specific strengths but suffer from trade-offs that limit their deployment [3,4]. Photonic approaches require complex integration [5,6]; MMIC-based solutions offer speed but suffer from insertion loss and limited power handling [7], and DMTLs provide compactness and bandwidth at the cost of switch count and dispersion [8,9,10].

RF MEMS switches offer a compelling alternative, combining low insertion loss, high linearity, and wide bandwidth with low static power consumption. Recent research has re-evaluated their potential in the context of mobile broadband, mmWave front ends, and satellite payloads [11,12,13] and suggests that RF MEMS are eminently suitable as the building blocks for future beamforming modules and networks. However, enhancements are needed, and research continues into new mechanical structures [14] (e.g., 3D origami design), novel materials [15] (e.g., silicon-on-insulator), improved packaging [16], and long-term reliability [17]. These developments renew interest in MEMS-based TDUs as viable components in advanced RF systems.

Parallel advances in beamforming architectures have also expanded the design space. Joint phase–time arrays (JPTAs), for example, propose hybrid analog front-ends that combine coarse TDU elements with fine-grained phase shifters for efficient beam control across frequency [18,19]. These systems increasingly demand compact, wideband, and low-loss TDU blocks compatible with low-cost PCB platforms.

Motivated by these trends and by the recent availability of a compact, reliable, and commercially packaged RF MEMS switch, this work presents a practical, manufacturable TDU implementation. The design leverages a switched-line architecture with 4-bit resolution over a 0–413 ps range, integrated on a standard multilayer PCB using conductor-backed coplanar waveguide (CBCPW) routing. In contrast to prior solutions based on custom MEMS dies, exotic substrates, or specialized assembly, this unit uses only off-the-shelf surface-mount components and industry-standard processes. It is, to our knowledge, one of the first demonstrations of a complete RF MEMS-based TDU using commercially available parts in a manufacturable, PCB-integrated format, offering a practical pathway from research to mass deployment. Measured results confirm broadband operation from 0.4 to 6 GHz, with an average insertion loss below 1.5 dB and group delay errors under 18.4 ps. These results support the validity of RF MEMS as a scalable solution for beamforming networks.

The main contributions can be summarized as (i) a commercially packaged, PCB-integrated 4-bit switched-line TDU spanning 0.4–6 GHz using only off-the-shelf components and low-voltage control; (ii) an analysis that reconciles simulation and measurement by using a multiport switch representation that includes the OFF-path termination network, identifying OFF-path-induced shunt loading as the dominant cause of the gap; and (iii) practical guidance for switched-line TDUs: when unused branches are terminated, include the complete OFF-path network in the model, or avoid the shunt, and prioritize interface matching to control residual ripple and loss.

In Section 2, the switched-line architecture and design rationale are described. Section 3 discusses the fabrication and assembly of the TDU PCB. Simulation and measurement results are presented in Section 4, and Section 5 compares prior comparable works and discusses broader implications, including a brief reconciliation of simulation and measurement results to clarify modeling assumptions.

## 2. TDU Design and Simulation

**Figure 1:**
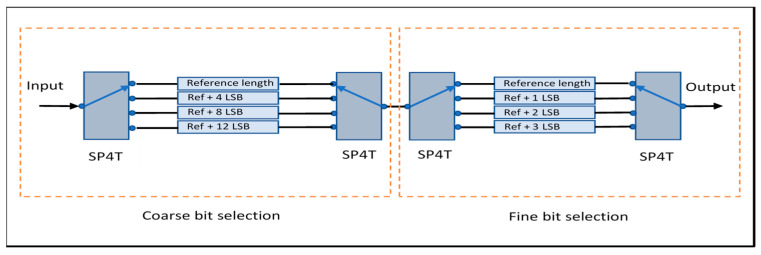
TDU schematic showing two 2-bit sections (course and fine bit selection).

The TDU developed in this work is based on a switched-line architecture formed by cascading two 2-bit sections, creating a compact 4-bit structure. Figure 1 illustrates the topology. A switched-line delay unit creates discrete time delays by selecting between transmission lines of varying lengths using RF switches. Each line introduces a fixed delay increment relative to a reference “zero” delay, achieving a maximum delay of 412.5 ps, with LSB = 27.5 ps.

The layout was simulated using a hybrid co-simulation approach. The PCB geometry, delay lines, and SMA ports were modeled in full 3D using Ansys HFSS, while the RF MEMS switches were represented by a lumped-element model.

**Figure 2:**
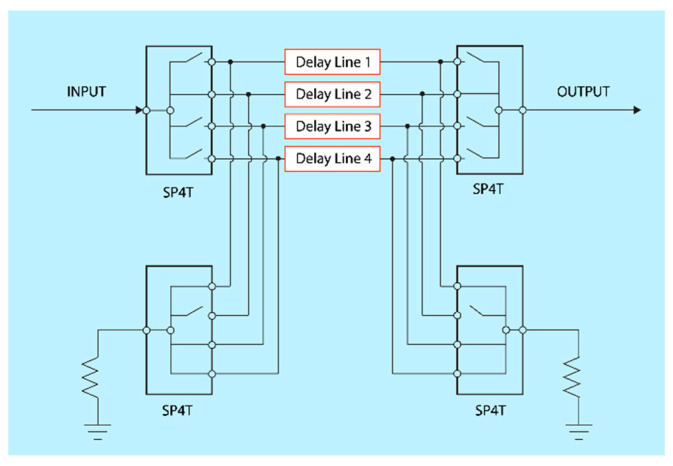
Terminating unused delay lines with RF MEMS switches for one 2-bit section.

A key challenge with switched-line delay topology is the risk of half-wavelength resonance in unused delay lines, which can lead to deep notches in the frequency response and significantly distort group delay ripple. Prior work has addressed this using shunt terminations [20], careful tuning of the reference delay length [21], or additional series switching networks [22]. This work investigated an approach in which unused paths were terminated to ground using separate shunt RF MEMS switches, as shown in Figure 2, to emulate broadband matched terminations. The impact of this termination strategy is evaluated using measurements and refined modeling in Section 5.

The switches used are MM5140 RF MEMS SP4T devices (Menlo Micro, Irvine, CA, USA) selected for their low insertion loss (0.5 dB at 8 GHz), 25 dB isolation at 6 GHz, SMT compatibility, and broadband performance up to 8 GHz [23]. These are LGA-packaged switches featuring integrated electrostatic drive circuitry, enabling low-voltage digital control without external switch activation hardware. The RF behavior of each switch was modeled using a lumped-element equivalent circuit derived from a circuit published by the manufacturer for a similar device.

Impedance matching between the RF MEMS switches and delay lines proved critical. Although simulations showed acceptable matching (better than −12 dB), later measurements indicated mismatch-related ripple and increased insertion loss in certain delay states (see Section 4). In practice, cascading four series switches magnifies the effect of small discontinuities. This highlights the sensitivity of switched-line architectures to even modest mismatch at transitions and port interfaces.

All delay lines are implemented using conductor-backed coplanar waveguide (CBCPW) on Rogers RO4003 laminate (0.508 mm thick, 1 oz copper). CBCPW was selected over microstrip and other planar structures due to its lower dispersion and better group delay flatness across wide bandwidths, as confirmed by our own full-wave simulations. While not identical, similar performance advantages have been observed in finite-grounded coplanar waveguide (FGCPW) structures. A comparative study using FGCPW transmission lines [24] concluded that coplanar waveguide topologies offer superior wideband performance over microstrip, supporting the selection of CBCPW for this work.

All coplanar waveguide (CPW) structures are sensitive to asymmetries and discontinuities, which can excite odd modes of propagation, leading to impedance imbalance and group delay distortion. Two accepted methods for suppressing parasitic modes are the use of air bridges or vias to a lower ground plane. This design employs vias for simplicity and compatibility with standard PCB processes.

CPW is also more sensitive to manufacturing tolerances than microstrip. Small deviations in line width or gap can result in substantial impedance variation. The layout was optimized for manufacturability using conservative spacing and smoothed transitions to minimize fabrication-induced mismatch.

**Figure 3:**
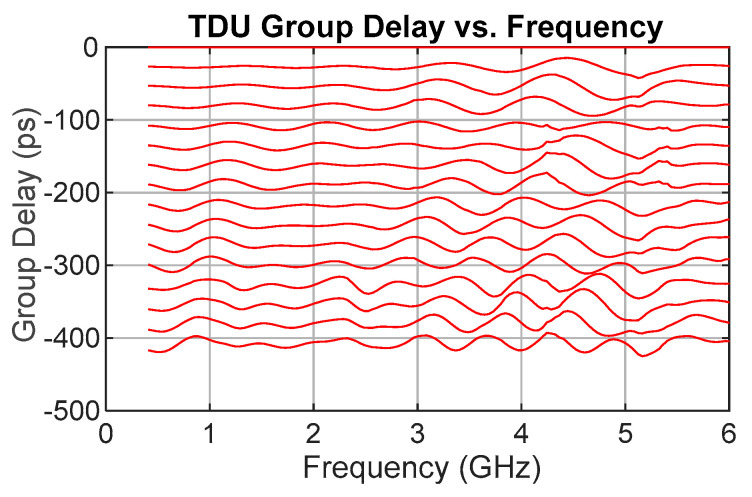
Simulation results showing TDU group delay for all 16 delay states.

Figure 3 shows the simulation results for the group delay of all 16 delay states. To quantify the performance, the mean absolute error (MAE) is used to measure the accuracy of the delay value, and the standard deviation indicates the amount of ripple about the mean. It is expressed as

(1) $$MAE=\frac{1}{N}\sum_{i=1}^{N}\left|t_{simulated}^{i}-t_{ideal}^{i}\right|$$

where N is the frequency sample size, $t_{simulated}$ is the simulated time delay, and $t_{ideal}$ is the ideal or design time delay.

Overall, for all values at all frequencies and all delay settings, the MAE is 5.56 ps, with a standard deviation of 6.78 ps. This indicates a very small accuracy error over the full delay ranges and frequencies with a small ripple such that 95% of values are within +/− LSB/2 of the desired value, i.e., within two standard deviations of the desired delay value.

**Figure 4:**
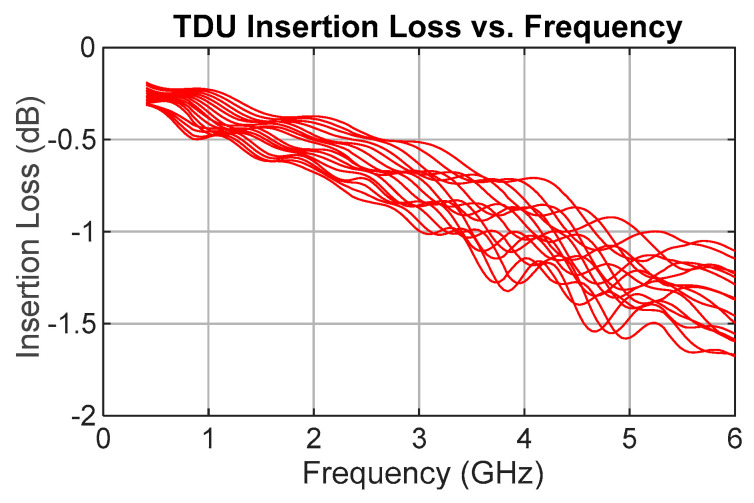
Simulation results showing TDU insertion loss (S21) for all 16 delay states.

Figure 4 shows the simulation results for the insertion loss. The average loss is 0.82 dB, with a maximum loss of 1.68 dB, within the frequency band of operation. At 6 GHz, the loss ranges from 1.21 dB to 1.68 dB, indicating that the difference in levels between zero delay and delay state 15 is expected to be just 0.47 dB. The simulation model for the switches indicated a loss per switch of 0.2 dB (times 4), giving a line loss range at 6 GHz of 0.41 dB to 0.88 dB.

**Figure 5:**
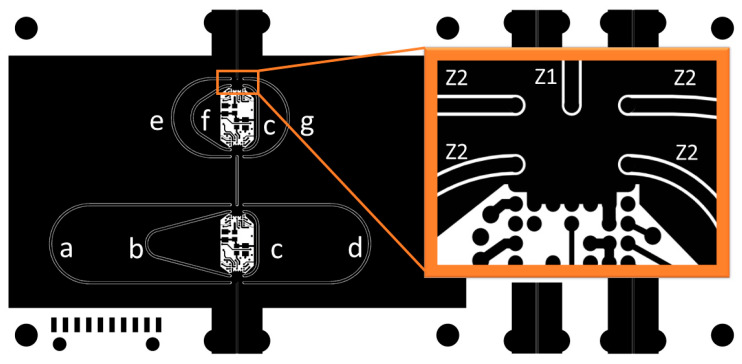
TDU delay line layout showing the 7 delay lines (a–g) and the tracks under the RF MEMS switch along with their characteristic impedances Z1 and Z2.

Figure 5 shows the delay line outline, and Table 1 summarizes the design lengths of each delay line. The input, output, and interconnect lines are designed to 50 Ω, whereas the delay lines use 54 Ω to balance return loss across 0.4–6 GHz when cascaded with switch parasitics. Table 2 lists the CBCPW dimensions for the 50 Ω (Z1) and 54 Ω (Z2) tracks.

## 3. Fabrication and Assembly

The TDU was implemented as a four-layer printed circuit board (PCB) with a layer of RF substrate (Rogers RO4003, 0.508 mm thick) for RF transmission and a layer of FR4 for mechanical support, DC power distribution, and control signal routing.

The series RF MEMS switches were mounted directly on the CBCPW tracks on the top side of the PCB, and the shunt RF MEMS switches were mounted on the bottom side directly under the transmission delay lines so as to keep the terminations as close as possible to the transmission lines. The SPI RF MEMS control lines were routed on the inner FR4 layer.

**Figure 6:**
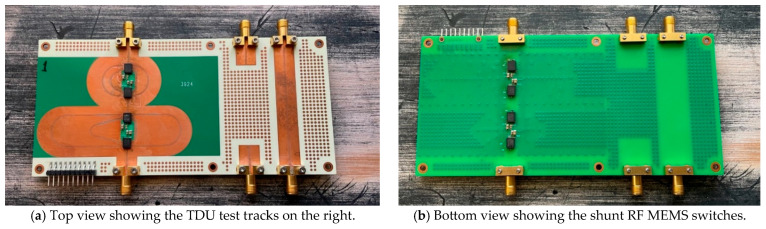
Photograph of the finished PCB.

The PCB was manufactured and assembled using standard PCB processes and surface mount soldering techniques, and the finished PCB is shown in Figure 6.

## 4. Measurement and Results

The results of the completed TDU are presented below, with de-embedding applied to remove the influence of input and output connectors and feed lines. Measurements were performed using a Keysight N5224 vector network analyzer (VNA) calibrated by the standard SOLT (Short-Open-Load-Through) method at the SMA connector reference plane.

The TDU measurements began with the characterization of a reference transmission line that was included on the same PCB to verify the expected impedance of the delay line geometry. The reference line was designed to provide a known 50 Ω path. When measured, the line exhibited a characteristic impedance of 55.5 Ω. The dominant source of this error was manufacturing tolerance. The track width measured 0.388 mm vs. 0.43 mm (design), and the gap measured 0.134 mm vs. 0.10 mm (design). These differences account for the impedance shift. For this PCB, the delay line impedances deviate from the 50 Ω target by 11%, slightly outside typical 10% PCB tolerances for RF lines.

**Figure 7:**
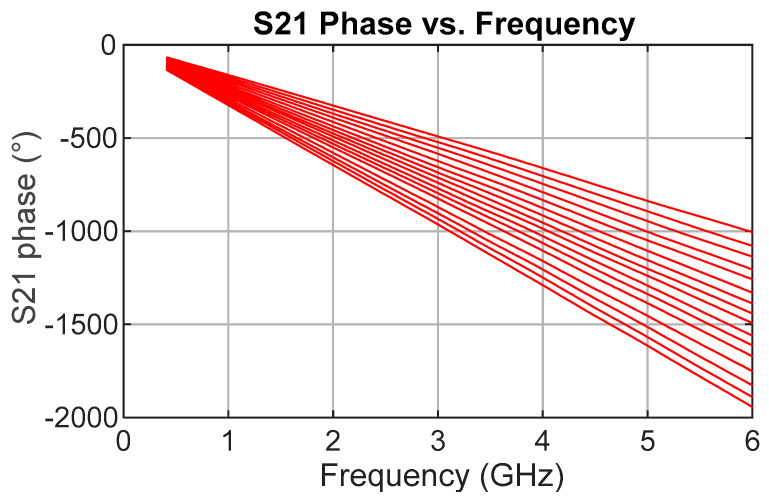
Measured results for the transmission phase (in degrees) for each of the 16 delay states.

The transmission phase response (S21) of the TDU was first analyzed to verify delay accuracy. Figure 7 shows the measured unwrapped phase versus frequency, exhibiting good separation between each delay state with no crossovers and a consistent relative phase shift. This confirmed the intended behavior of the switched-line topology and the consistent timing alignment of signals passing through each state. Precise measurements for each delay state show a general excess delay, on average 6% greater than design, indicating the transmission lines are slightly longer than required. The average delay step size is 29.0 ps compared to the design value of 27.5 ps.

**Figure 8:**
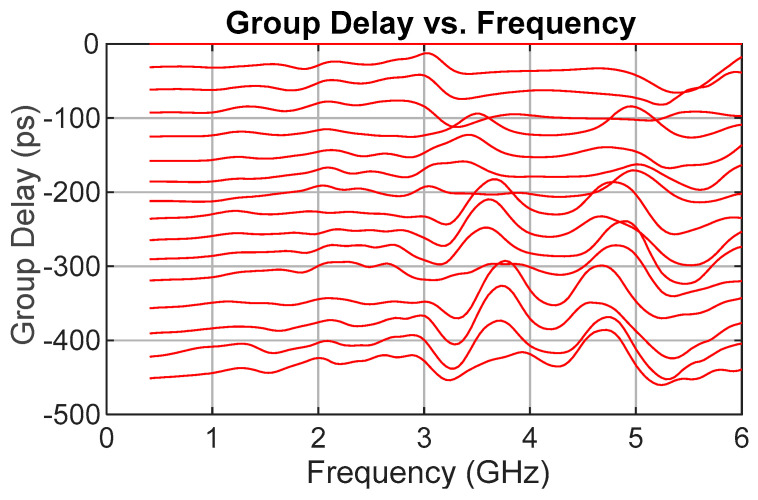
Measured group delay for all TDU delay states in picoseconds.

While S21 phase confirmed correct operation, group delay, calculated as the negative derivative of phase with respect to frequency, was the primary figure of merit for TDU performance. Figure 8 presents measured group delay versus frequency for all delay states relative to the reference or “zero” path. Each delay is clean to about 3 GHz, after which ripple begins to appear. A distinct grouping is evident. The first four states (coarse “zero” plus fine bits) share a similar dip from roughly 3 to 5.5 GHz, and the next four (through the 4-LSB coarse line) show a narrower dip around 3.5–5 GHz. This pattern repeats, implicating the coarse-bit delay lines as the dominant contributor to ripple.

Again, the MAE and standard deviation are used to quantify the performance. Across all delay states and frequencies, the overall MAE was 18.4 ps, with a standard deviation of 13.3 ps, as compared with an MAE of 5.56 ps and a standard deviation of 6.78 ps in the simulation. The values for each delay step are given in Table 3.

**Figure 9:**
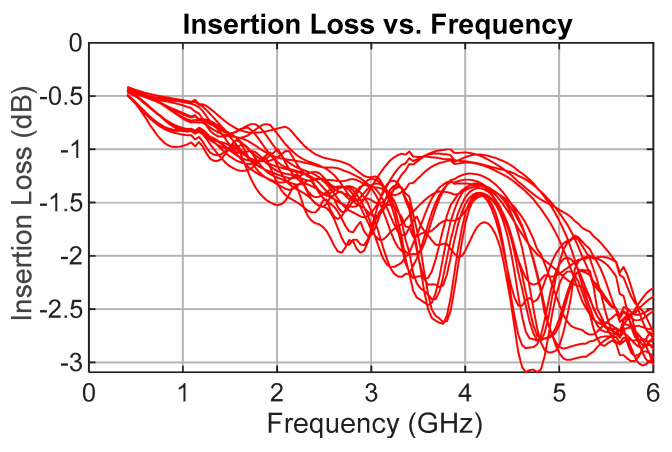
Measured insertion loss (S21 dB) for all TDU delay states.

Insertion loss was the second most critical parameter. As shown in Figure 9, the measured insertion loss (S21), including the reference lines, ranges from 2.3 dB to 3 dB at 6 GHz for all delay states, with 3.09 dB as the worst-case value across all frequencies and delay configurations. According to the manufacturer’s measured S-parameters, the MM5140 switch exhibits an insertion loss of 0.44 dB at 6 GHz, giving a total of 1.76 dB for the four switches in each signal path. This leaves the delay line losses ranging from 0.54 dB to 1.24 dB.

The average insertion loss across all frequencies and delay states is 1.5 dB, with a standard deviation of 0.67 dB.

**Figure 10:**
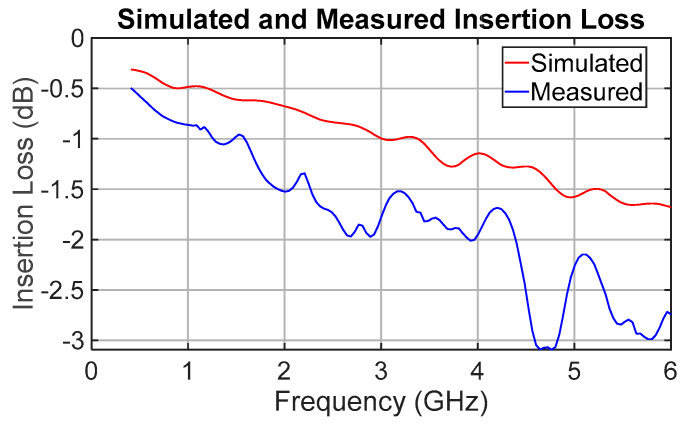
Comparison of measured and simulated S21 (dB) for the longest delay.

To further illustrate the difference between simulation and measurement, Figure 10 overlays the simulated and measured insertion loss for delay state 15. The measured result follows the same general trend but shows greater attenuation and greater ripple. The underlying causes of these differences between simulation and measurement are analyzed in detail in Section 5.1, where a refined multiport switch model is shown to accurately explain all measured features.

**Figure 11:**
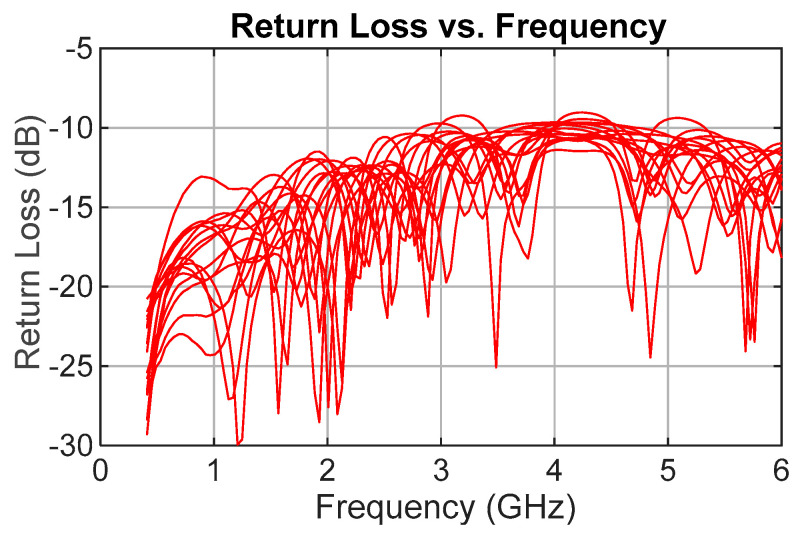
Measured return loss (S11 dB) for all TDU delay states.

Return loss (S11) measurements, the third key parameter, are presented in Figure 11. The measured return loss remains better than 9 dB across the frequency band of interest compared with the simulated result of approximately 12 dB, which represents the design expectation.

Table 3 summarizes the measured performance by delay state. We see that, over the complete operational band, the per-state insertion loss spans 2.68 to 3.09 dB (≈±0.21 dB about the mean), and the delay error exhibits a consistent positive bias of 5–36 ps.

## 5. Discussion and Comparison with State of the Art

**Figure 12:**
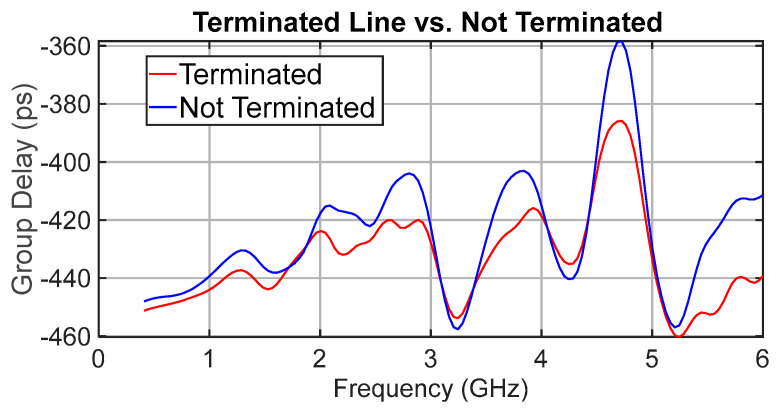
Comparison of group delay for terminated and unterminated unused delay lines for state 15 delay.

A key hypothesis of this research was that terminating unused delay tracks would eliminate half-wave resonances. Figure 12 shows a comparison of the group delay for delay state 15 (the longest delay path). There is a measurable benefit from terminating the unused tracks: the peak delay variation improves by approximately 25 ps at 4.7 GHz (nearly one LSB). However, the residual ripple with terminations remains 74 ps peak to peak, so the overall benefit is moderate. As shown later in Section 5.1, this residual ripple originates from the shunt loading of the OFF-state switch ports rather than incomplete termination.

Other delay values exhibited similar, though smaller, improvements. For example, the zero delay shows a 7 ps improvement around one frequency.

Early circuit performance simulations, with unterminated unused tracks, indicated severe group delay distortions of up to 190 ps at frequencies equal to the resonant frequency of those unused tracks. As this delay variation is nearly seven times the LSB, the initial strategy was to terminate unused tracks to suppress resonances. However, the measured results suggest that, with careful PCB layout, the impact of the unterminated tracks can be mitigated.

The conclusion is that, with prudent use of grounding vias to suppress coupling between delay lines and the use of confined transmission line structures such as CBCPW, acceptable performance can be achieved without termination. In this implementation, better impedance matching would likely have delivered greater performance improvements than shunt terminations. These results highlight a key insight: while termination is a straightforward solution to suppress resonances, the root cause lies in electromagnetic coupling and reflection paths within the layout. In this work, the use of CBCPW, together with copper-filled vias connecting the top and bottom grounds, effectively reduced coupling between active and inactive delay lines by laterally confining the electromagnetic fields. This configuration forms a channelized structure in which the finite lower ground and via sidewalls prevent return-current crossover and surface-wave propagation between neighboring paths. These are well-established microwave design techniques [23]. The combined benefits of CBCPW geometry and grounding vias were confirmed by full-wave EM simulation. This suggests that careful electromagnetic design can serve as a viable alternative to termination in similar switched-line systems, simplifying the layout and reducing component count.

### 5.1. Simulation-Measurement Reconciliation

A brief reconciliation explains the simulation-to-measurement gap.

The initial co-simulation used a lumped switch abstraction and published 2-port data for the switch, which was too simplistic. Published 2-port S-parameters for multiport devices are reduced under the assumption that all unused ports are terminated in the characteristic impedance Zo (typically 50 Ω). In this design, the unused MM5140 throws are connected to a coplanar line that continues into a shorting network implemented with a parallel MM5140. Consequently, the two-port representation of the active throw does not capture the port-environment-dependent loading present on the board. This discrepancy explains why the initial EM plus 2-port co-simulation predicted substantially better return loss and insertion loss than those measured. That is to say, while the design in Figure 2 terminates each unused delay line to stop any resonances, the active delay line has the added shunt load of an open port on a parallel switch, and this load is material to performance and cannot be neglected.

To resolve this, a compact reciprocal, passive 5-port macro was built for the MM5140 and embedded into the complete nodal model of the TDU so that each switch throw “sees” its actual board environment. The switch OFF state is not an open circuit. The MM5140 OFF path is essentially a capacitive ladder: big pad caps to ground on both pads (circa 300 fF) with a small series coupling cap between common to throw (12 fF), plus the common side is terminated in 50 Ω. It is a π network to ground and presents a very low impedance circuit to ground at 8 GHz.

**Figure 13:**
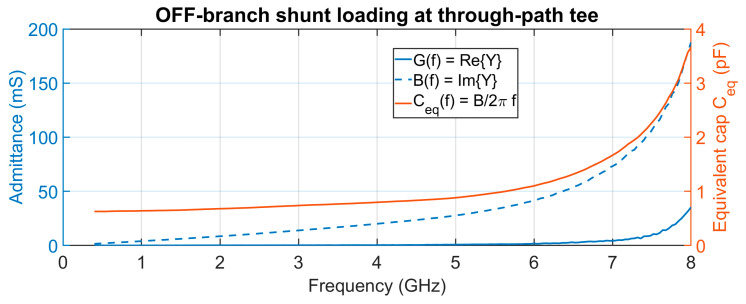
Equivalent shunt admittance (mS) at the through-path tee (includes via segment) and the equivalent capacitance (pF) of the load.

Figure 13 shows the equivalent shunt admittance B(f) at the through-path tee, including the via connection, to the OFF-path termination switch. The extracted equivalent capacitance is about 600 fF at 0.4 GHz, roughly twice the nominal 300 fF pad capacitance of a single OFF port. This occurs due to the short via linking the two switches. Without the via, the equivalent capacitance is about 450 fF. This additional shunt load exacerbates the passband ripple, as observed in the measured results.

A sensitivity check was also performed by varying the dielectric loss tangent of the RO4003 substrate. Increasing tan δ from zero to values twenty times above the specified value affected only the insertion-loss slope (negatively) and return loss (positively), while the group-delay RMS ripple actually improved by a small amount (4 ps). This confirms that dielectric loss degrades transmission magnitude but has negligible influence on the ripple, which instead arises from impedance discontinuities and OFF-path loading at the switch junctions.

With the 5-port macro and the full OFF-branch network included, the model closely tracks measurements: RMS errors of 2.5 dB (|S11|), 15° (∠S11), 1.1 dB (|S21|), 6° (∠S21), and 47 ps (group delay).

The strong, dispersive shunt admittance introduced by the loading of the OFF port of the parallel switch (a) worsens the return-loss null structure, (b) adds excess insertion loss, and (c) produces the measured group-delay ripple peaks via multi-path internal reflections around the branch.

The systematic impedance bias of 55.5 Ω instead of 50 Ω is a secondary mechanism. This creates a per-junction reflection coefficient of order Γ = (Zc − Zo)/(Zc + Zo) ≈ 0.05 (−26.5 dB). With four switches and several line segments, these small discontinuities set a baseline ripple in S11/S21 and group delay, but they are not the dominant cause relative to the switch OFF-path shunt admittance.

Subsequent MATLAB (R2023a) analysis with the perfect-switch model confirms that the group-delay ripple increases by roughly 15 ps RMS when the delay-line impedance is varied from 50 Ω to 60 Ω, corresponding to a sensitivity of approximately 1.5 ps RMS per ohm of impedance deviation.

Using the corrected model to explore alternatives indicates that removing the parallel shorting switches and tightening interface matching can achieve return loss > 15 dB, insertion loss ≈ 0.9 dB at 6 GHz, and RMS group delay ripple ≈ 6.7 ps.

**Figure 14:**
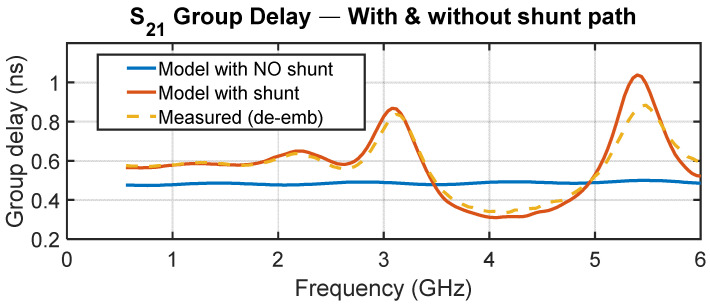
Group delay for state 0: measurement vs. corrected multiport model with the OFF-path shunt and a counterfactual model without the shunt. The corrected model aligns with measurement; removing the shunt predicts much lower ripple, isolating the OFF-path loading as the primary mechanism.

Figure 14 illustrates the corrected multiport model for the absolute delay state 0 over 0.4–6 GHz. The curve with the OFF-path shunt network (parallel switch plus termination) closely reproduces the measured group delay, while a hypothetical variant without the shunt shows the substantial ripple reduction achievable when the shorting network is eliminated. This isolates the OFF-path shunt as the dominant contributor to the observed ripple; layout-related reflections set only a smaller baseline.

### 5.2. Comparison with Prior Works

Four published works were selected for direct comparison with this research. While none of these use a PCB with commercially packaged RF MEMS switches, each provides a relevant benchmark in terms of delay architecture, frequency range, and performance metrics. Papers relying on monolithic integration (e.g., silicon, LCP, alumina) or MMIC switches were excluded due to architectural differences.

Table 4 summarizes the key attributes of these comparable works, including delay range, insertion loss, and figure of merit (FOM).

Authors of [24] report the first integration of packaged RF MEMS switches on a PCB in a switched-line TDU. The MEMS devices were hermetically sealed dies that required wire bonding, introducing parasitic losses.

The work in [25] demonstrates the first LGA-packaged RF MEMS on a PCB. However, it exhibited over 100 ps of group delay ripple on a nominal 49 ps delay, indicating significant impedance and control issues.

The design in [26] uses metal-contact MEMS in a hermetically sealed die mounted on a PCB, achieving 77 ps delay over a 1.7–2.7 GHz band. This design requires 90 V for actuation and suffers parasitic losses due to wire bonding.

The work [27] sets a high benchmark for broadband operation (6–12 GHz), with a delay of 3255 ps. However, the MEMS switches are custom and not commercially available, require 90 V activation, and involve 20 switches with up to 16 dB insertion loss. Its FOM is 203 ps/dB but comes with considerable complexity.

The TDU presented in this paper achieves an FOM of 152.8 ps/dB, noteworthy given its reliance on commercially available components, low-voltage actuation, and standard PCB manufacturing. This fulfills the original goal of demonstrating a manufacturable, broadband TDU with practical packaging and control and appears to be the first report of using a commercial off-the-shelf packaged RF MEMS switch in this architecture.

Practical takeaways include (i) maintaining return loss better than about 15 dB to preserve delay flatness; (ii) prioritizing matching at switch–line interfaces; (iii) accounting for CPW tolerance sensitivity; and (iv) using multiport switch models when OFF-path terminations are present.

## 6. Conclusions

This work presents the design, fabrication, and experimental validation of a 4-bit switched-line true time delay unit (TDU) using commercially packaged RF MEMS switches and standard multilayer PCB processes. The prototype operates from 0.4 to 6 GHz, with a maximum delay of 413 ps and a 4-bit resolution, achieving a figure of merit (FOM) of 152.8 ps/dB. Key performance metrics include an average insertion loss of 1.5 dB and a mean absolute delay error below 18.4 ps across all states.

The novelty of this work lies in its use of commercially available RF MEMS switches in a compact, manufacturable PCB implementation. Unlike prior designs that required custom dies, high-voltage switch actuation, or exotic substrates, this TDU leverages LGA-packaged RF MEMS switches and coplanar PCB technology to achieve high performance with low complexity.

Subsequent analysis with a corrected multiport switch model shows that OFF-path termination-induced shunt loading is the dominant cause of the simulation–measurement gap. Once this mechanism is modeled or removed, the remaining ripple is explained by interface mismatch and small layout reflections, providing clear guidance for refinement.

A brief reconciliation (Section 5) shows that incorporating a compact multiport switch model and the OFF-path termination network brings simulation and measurement into close agreement.

The findings show that high-performance TDUs can be achieved without custom fabrication, paving the way for deployable RF MEMS-based phased array systems. Such TDUs are essential for enabling beam squint-free, dispersion-free wideband operation in advanced array systems, including those targeting 6G, satellite communication, radar, and mobile platforms.

While the demonstrated prototype meets its design objectives, several practical limitations should be acknowledged. The overall footprint is larger than that of monolithic or MMIC solutions, and the performance remains sensitive to fabrication tolerances and parasitic loading of packaged switches. Nevertheless, the design offers the advantages of high power handling, negligible DC consumption, and full compatibility with standard PCB processes. Future refinements could include multilayer or fractal delay-line geometries to reduce area, improved impedance control through laser trimming and tighter process tolerances, and the use of lower-parasitic switch packaging. The measured group-delay ripple can be further reduced by accounting for the multiport loading effects identified in Section 5.1 and by optimizing impedance matching at switch-line junctions. A further opportunity lies in combining true time delay with phase control for joint phase–time adjustment (JPTA), enabling fine-grain beam steering in next-generation wideband arrays.

## Figures and Tables

**Table 1:** Delay line design lengths.

Delay Line	Length (mm)	Delay Line	Length (mm)	Delay Line	Length (mm)
a	88.14	d	64.57	f	23.32
b	41.00	e	35.10	g	29.21
c	17.43				

**Table 2:** CBCPW track impedances and dimensions.

Track	Impedance (Ω)	Track Width (mm)	Gap Width (mm)
Z1	50	0.43	0.10
Z2	54	0.39	0.12
RO4003C, h = 0.508 mm, t = 1 oz.			

**Table 3:** Summary table of measured TDU performance results for every delay state.

Delay State or Summary	Average Delay (ps)	Target Delay (ps)	MAE (ps)	Delay Std Dev (ps)	Average Loss (dB)	Max Loss (dB)
0	0.0	0	0.0	0.0	1.24	2.68
1	−32.6	−27.5	8.9	10.1	1.22	2.73
2	−60.4	−55	8.9	8.5	1.30	3.02
3	−89.8	−82.5	10.1	6.9	1.31	2.77
4	−122.3	−110	12.9	8.5	1.54	2.87
5	−156.8	−137.5	19.6	7.6	1.49	2.97
6	−185.7	−165	21.1	8.2	1.50	2.97
7	−209.5	−192.5	16.7	10.1	1.54	2.94
8	−235.0	−220	14.8	8.8	1.55	2.76
9	−263.1	−247.5	15.4	9.3	1.51	2.75
10	−290.3	−275	15.3	8.5	1.56	2.89
11	−313.4	−302.5	12.9	10.7	1.54	2.72
12	−357.1	−330	25.7	9.9	1.68	2.89
13	−392.5	−357.5	34.2	11.5	1.64	2.86
14	−421.0	−385	35.6	12.0	1.70	2.98
15	−435.6	−412.5	23.6	16.2	1.70	2.99
Overall			18.4	13.3	1.52	3.09

Note: Negative delay values indicate that the propagation time of the selected path is greater than that of the zero-delay reference path. The ‘Overall’ values represent the aggregate metrics computed across all delay states and frequencies.

**Table 4:** Comparison of results with state-of-the-art.

Reference	[24]	[25]	[26]	[27]	This Work
Year	2013	2014	2015	2016	2025
Technology	Packaged MEMS and PCB	Packaged MEMS and PCB	MEMS and PCB	MEMS and PCB	Packaged MEMS and PCB
TX line technology	microstrip	CPW	microstrip	microstrip	CBCPW
Switch	MEMS	MEMS	MEMS	MEMS	MEMS
Frequency (GHz)	8–12	0–5	1.7–2.7	6–12	0.4–6
Bits	5	3	4	5	4
Max time delay (ps)	96.7	49	77	3255	472
Loss average (dB)	3.1	2.6 *	0.57 *	12	1.5
Loss maximum (dB)	4.7 *	7	0.9	16	3.09
Time error average (ps)	0.6	58 *	-	-	2.54
Time error maximum (ps)	1.2	100 *	2.06	3.2 (max at center freq.)	10.56
Return Loss (dB)	15	7	13.4	12	9
Loss per bit (dB)	0.62	2.333333	0.225	3.2	0.7725
FOM ps/dB loss	20.6	7.0	85.6	203.4	152.8

* Value read from graphic and not explicitly stated by author.

## Data Availability

Data are contained within the article.
